# Contrasted Patterns of Selection on MHC-Linked Microsatellites in Natural Populations of the Malagasy Plague Reservoir

**DOI:** 10.1371/journal.pone.0032814

**Published:** 2012-03-05

**Authors:** Charlotte Tollenaere, Svilena Ivanova, Jean-Marc Duplantier, Anne Loiseau, Lila Rahalison, Soanandrasana Rahelinirina, Carine Brouat

**Affiliations:** 1 Institut de Recherche pour le Développement, UMR CBGP (INRA/IRD/Cirad/Montpellier SupAgro), Montferrier sur Lez, France; 2 Institut National de la Recherche Agronomique, UMR CBGP (INRA/IRD/Cirad/Montpellier SupAgro), Montferrier sur Lez, France; 3 Institut Pasteur de Madagascar, Unité Peste, Antananarivo, Madagascar; University of Lausanne, Switzerland

## Abstract

Plague (*Yersinia pestis* infection) is a highly virulent rodent disease that persists in many natural ecosystems. The black rat (*Rattus rattus*) is the main host involved in the plague focus of the central highlands of Madagascar. Black rat populations from this area are highly resistant to plague, whereas those from areas in which the disease is absent (low altitude zones of Madagascar) are susceptible. Various lines of evidence suggest a role for the Major Histocompatibility Complex (MHC) in plague resistance. We therefore used the MHC region as a candidate for detecting signatures of plague-mediated selection in Malagasy black rats, by comparing population genetic structures for five MHC-linked microsatellites and neutral markers in two sampling designs. We first compared four pairs of populations, each pair including one population from the plague focus and one from the disease-free zone. Plague-mediated selection was expected to result in greater genetic differentiation between the two zones than expected under neutrality and this was observed for one MHC-class I-linked locus (D20Img2). For this marker as well as for four other MHC-linked loci, a geographic pattern of genetic structure was found at local scale within the plague focus. This pattern would be expected if plague selection pressures were spatially variable. Finally, another MHC-class I-linked locus (D20Rat21) showed evidences of balancing selection, but it seems more likely that this selection would be related to unknown pathogens more widely distributed in Madagascar than plague.

## Introduction

Immune genes have been shown to be strongly affected by natural selection [1], [2]. In particular, the genes of the Major Histocompatibility Complex (MHC) have attracted the attention of evolutionary biologists, because of their extraordinary polymorphism and fundamental role in vertebrate immunity [3]. This genetic complex contains three gene subfamilies [4]. Class I genes are expressed by nucleated somatic cells and play an essential role in defense against intracellular pathogens, as their products present antigens derived from infected cells to cytolytic T-cells. Class II genes are expressed by antigen-presenting cells of the immune system and encode receptors presenting peptides mostly derived from extracellular pathogens to T helper cells. However, the functional difference between these two classes is not clear-cut, as both MHC class I and II may be involved in recognizing endogenous and exogenous antigens [5]. Finally, MHC class III includes a diverse array of structurally unrelated genes, including several involved in innate immunity (e.g., proinflammatory cytokines, complement components).

Pathogens are thought to be one of the main selective forces acting on the MHC genes of their hosts [3], [6]. Pathogen-mediated balancing selection is generally thought to account for the tremendous diversity observed at MHC loci, but the underlying mechanisms remain debated [6], [7]. Three main hypotheses have been proposed: heterozygote advantage, rare-allele advantage and spatio-temporal variation of selective pressures [7]. Pathogen selection pressure may also drive a decrease in genetic diversity at MHC markers through directional selection, with the spread of an advantageous allele (positive selection), or selection against disadvantageous alleles (negative or purifying selection) [8]. Directional selection may occur if a single pathogen strain or species is thought to exert a major selective pressure on the host species considered (see for example [8]). Various studies have shown that natural selection acts on MHC genes and have investigated its underlying mechanisms by comparing the population genetic structure of MHC genes with that of neutral loci (see for example [9], [10]; reviewed in [6]). Some of these studies have suggested that variability in pathogen exposure acts as a selective pressure on MHC loci [11], [12], but most did not include descriptions of the geographic distribution of pathogen prevalence (but see [13]).

Two main technical approaches are used to study MHC variation. First, gene sequences can be analyzed directly (using classical, or next-generation sequencing methods [14], [15] or by using a sequence library, e.g., by SSCP _*Single Strand Conformation Polymorphism*_ [16]). Second, MHC-linked microsatellites may be used, as linkage disequilibrium may stamp loci close to MHC genes with similar signatures of selection [17]. This latter approach has the advantages of allowing the analysis of several MHC loci at a moderate cost. It also allows the use of comparable markers (e.g., microsatellites) for the inference of population genetic structure and detection of signatures of selection [7]. Gene-linked microsatellites have already been shown to be useful for detecting natural selection acting on MHC genes (see for example [17]–[19]) or other candidate genes [20], [21]. The reliability of this indirect approach has been assessed by the observed correlation between the allelic variation of microsatellites and neighboring genes [22] and by the reported association between candidate gene-linked microsatellite diversity and parasite load [23].

Plague (*Yersinia pestis* infection) is among the most virulent of known pathogens for humans and susceptible rodents, resulting in high mortality rates in natural populations [24], [25]. Plague epidemics may therefore exert strong selective pressure on host immune genes. Resistance phenotypes may evolve rapidly in reservoir populations, as suggested by the association between the occurrence of plague and plague resistance reported for *Onychomys leucogaster* in North America [26] and *Mastomys natalensis* in South Africa [27]. This would also be the case for the black rat (*Rattus rattus*), the main reservoir of plague in Madagascar [28].

Plague was first reported in Madagascar in 1898. Areas of the central highlands of this country, at elevations above 800 meters have represented a plague focus since the 1920s, with hundreds of human cases reported each year and circulation within rodent populations. By contrast, there is no evidence of plague circulation in rural areas of the lowland zone of Madagascar [28], [29]. The black rat is usually considered to be highly susceptible to plague [24]. However, the *R. rattus* populations from the Malagasy plague focus (central highlands) are much more resistant (LD_50_ 1000 times higher) than black rat populations from plague-free zones (low altitude regions) [30], [31]. This resistance is transmitted to laboratory-born descendants [30], suggesting a genetic basis.

MHC genes may be involved in the genetics of plague resistance. Indeed, a recent quantitative genetics study showed the presence of a resistance locus (which remains to be precisely identified) within the MHC region in the laboratory mouse [32]. In addition, various laboratory studies have demonstrated the potential importance of MHC products for the immune response to plague. Indeed, *Y. pestis* epitopes have been shown to be recognized by MHC class II receptors of the laboratory mouse [33], as expected for this mostly extracellular pathogen [34]. However, MHC class I molecules may also be involved, as human cytolytic T cells are stimulated during *Y. pestis* infection [35]. Finally, the Tnf-α (*Tumor necrosis factor*) gene, an MHC class III gene, encodes the proinflammatory cytokine TNF-α, which has been shown to protect against *Y. pestis* experimental infection in laboratory mice ([36], [37], see also [38]).

**Figure 1:**
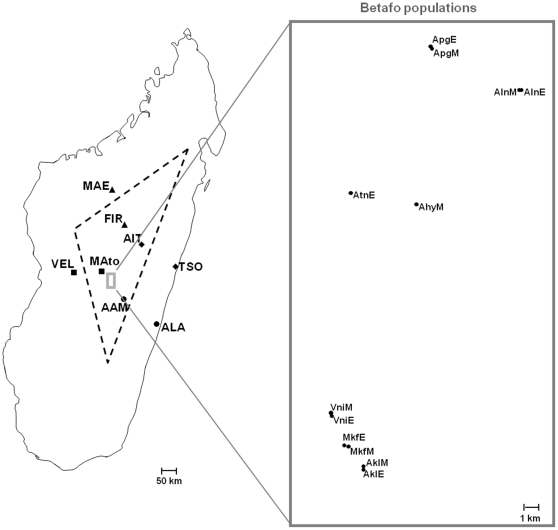
Location of the sampled sites. For the Madagascar dataset, each population pair, consisting of one population from the plague focus and one from the plague-free zone, is identified by a different symbol. The principal Malagasy plague focus is outlined by a black dotted line and the region of Betafo is outlined in grey.

This study uses the MHC gene family as candidate for plague resistance in wild populations of Malagasy *R. rattus*. In addition to shedding light on the theoretical question of the way in which selection shapes MHC variation in wild rodents, this research is a step towards a better understanding of plague circulation in the central highlands of Madagascar, which remains one of the most important plague foci in the world [24], [39]. To this purpose, we used a population genetics approach based on the comparison of MHC-linked and neutral microsatellites in two contrasting population designs. The first dataset focuses on four pairs of populations, each consisting of one population from within and another from outside the plague focus of Madagascar (Figure 1 and Table 1). In this so-called “Madagascar” dataset, plague-driven divergent selection within these pairs of populations may imply higher levels of genetic differentiation at MHC markers than at neutral markers [3]. However, the presence of plague is not the only selective environmental factor characterizing the Malagasy plague focus. Elevation and the ecological conditions typical of regions more than 800 m above sea level may also have an effect. We thus also used a metapopulation sampling within a restricted area of the plague focus in which human cases of plague are reported annually (Figure 1 and Table 1). As plague is likely to exert a uniform selection pressure on the *R. rattus* populations of this area, the level of genetic differentiation at MHC markers found to be under divergent selection in the Madagascar dataset would be expected to be lower than that for neutral markers in the so-called “Betafo” dataset [40]. Alternatively, spatial variation in plague selection pressure may result in more marked geographic patterns of genetic differentiation at these markers than at neutral markers [3].

**Table 1:** List of the populations analyzed in this study.

MADAGASCAR DATASET					
Population name	Distance(km)	Zone	Sampling year	Sample size	H_E_ (13 presumed neutral markers) ± S.E.
AAM	140	C.H.	2000	28	0.714±0.138
ALA		L.A.	1999	27	0.750±0.125
AIT	139	C.H.	2006	22	0.717±0.118
TSO		L.A.	2007	22	0.723±0.144
FIR	128	C.H.	1999	28	0.695±0.165
MAE		L.A.	1999	21	0.706±0.185
MAto	103	C.H.	1996	28	0.681±0.170
VEL		L.A.	2000	22	0.697±0.128

BETAFO DATASET			
Population name	Village	Sample size	H_E_ (13 presumed neutral markers)
ApgE	Amparihimboahangy	15	0.663±0.182
ApgM		20	0.687±0.142
AlnE	Ambatomilona	15	0.667±0.213
AlnM		20	0.670±0.189
AtnE	Antanetikely	14	0.727±0.165
AhyM	Ambatolahy	20	0.693±0.171
VniE	Vinanisoa	20	0.671±0.137
VniM		20	0.705±0.171
MkfE	Miraikofeno	14	0.717±0.163
MkfM		20	0.704±0.206
AklE	Ankelaka	20	0.660±0.158
AklM		16	0.715±0.137

Sample sizes and genetic diversity (expected heterozygosity: H_E_) are shown. C.H.: central highlands; L.A.: lowland areas.

## Results

### Studied loci and within-population analyses

Five MHC-linked microsatellite markers designed on the basis of genome sequence of *R. norvegicus* were analyzed, each corresponding to one of the three MHC classes [4] (Table 2). These five MHC-linked loci comprised two (Msat-Tnf and D20Img2) present as single copies (maximum of two alleles per individual), and three duplicated loci: RT1N1 and D20Rat41 were present as two copies and D20Rat21 was present as three copies at least (the locus D20Rat21 was also found to be duplicated in *R. norvegicus* [41], but no such information was found for RT1N1 and D20Rat41). A set of 13 previously described microsatellites (see methods) was used as a control for non-selective factors and is referred to as “presumed neutral microsatellites”.

**Table 2:** Characteristics of the five MHC-linked microsatellites loci analyzed in this study.

Class	Locus	Primer sequences	Closest gene	Repeat	Total number of alleles per locus	Number of alleles per ind.
		(primer reference or design method)	(distance)		(size range)	
**I**	**D20Rat21**	5′-CTGTGCTATGGCAGGAGATT-3′	RT1-M6 (500 bp)	TG and AG	42	2–6
		5′-GCCATCTTCAGCACTACAGG-3′			(289–359)	
		(Design on *R. norvegicus* genome)				
	**RT1N1**	5′-TCTCGTGGAATAGGCAGA-3′	RT1N1 (1500 bp)	AG	16	1–4
		5′-TGGCTGTCCTAGAACTCACT-3′			(302–375)	
		(Design on *R. rattus* sequences)				
	**D20Img2**	5′-CTGAGCTCCCTAGGACCTACAT-3′	Ddr1 (42000 bp)	CA	7	1–2
		5′-TCTCTTGTGTCAGGCTAATTAC-3′			(277–333)	
		[60]				
**III**	**Msat-Tnf**	5′-ACATAGGCATGGTGTCTCTG-3′	Tnf (300 bp)	CA	16	1–2
		5′-CAGGATTCTGTGGCAATCTG-3′			(147–180)	
		(Design on *R. rattus* sequences)				
**II**	**D20Rat41**	5′-AGTYCTCTTCTGGYCTCCAT -3′	RT1-Bb (4 400 bp)	TG	42	1–4
		5′- TGGGACGATGTGTCATATCC -3′			(162–220)	
		(Design on *R. rattus* sequences)				

The five loci are ranked as on the chromosome, with D20Rat21 the closest to the telomere.

The neighboring genes indicated are based on the *R. norvegicus* genome sequence.

Linkage disequilibrium (LD) and Hardy-Weinberg (HW) tests were performed on non duplicated MHC-linked and presumed neutral microsatellites, for the both datasets. LD tests gave significant results after correction for multiple testing in 15 of 2087 cases. These significant tests accounted for only 0.7% of the tests carried out and concerned various locus pairs and populations. The 15 non duplicated loci were thus considered independent. HW tests indicated significant heterozygote deficiencies for some loci (Rr67, D11R56, D11M5, Msat-Tnf) that can be explained by null alleles following Micro-checker. Mean null alleles frequencies estimated using FreeNA over the 20 populations were however lower than 0.05 for these loci (table S1), having therefore little chance to bias genetic differentiation estimates [42], and thus outlier detection. None of the HW tests for heterozygote excess were significant for the two non duplicated MHC-linked loci: Msat-Tnf and D20Img2.

### Detecting signatures of selection for the Madagascar dataset

For the Madagascar dataset, two model-based approaches were used to search for selection signatures (Fdist2 [43] and DetSel [44], [45]) in the two non duplicated MHC-linked and the 13 presumed neutral microsatellites. For MHC-linked loci, D20Img2 alone had a higher *F_ST_* than expected under neutrality in some population pairs, in both Fdist2 (AIT/TSO: *p* = 0.003; marginally significant probability for FIR/MAE: *p* = 0.052) and DetSel (AIT/TSO: *p* = 0.018; AAM/ALA: *p* = 0.02) (Table 3 and figures S1 and S3). Msat-Tnf was not detected as outlier in any comparison (Table 3 and figures S1 and S2). However, a few presumed neutral loci were found to have significantly higher *F_ST_* in some population pairs (see details in Table 3).

**Table 3:** Results of population differentiation tests for the Madagascar dataset analyses.

		Population pair			
		AAM-ALA	AIT-TSO	FIR-MAE	MATo-VEL
**Fdist2 analysis**	Msat-Tnf	0.441	0.226	0.272	0.457
	D20Img2	0.167	0.003	0.052	0.207
	Supposed neutral loci revealing significant (*p-value*)	Rr067 (*0.017*) D11R56 (*0.006*)	D10R20 (*0.045*)	Rr067 (*0.026*) D7R13 (*0.023*)	Rr014 (*0.005*) Rr054 (*0.011*)
**DetSel analysis**	Msat-Tnf	0.61	0.41	0.84	0.49
	D20Img2	0.024	0.018	0.102	0.159
	Supposed neutral loci revealing significant (*p-value*)	D11R56 (*0.003*)	Rr093 (*0.003*)	D10R20 (*0.049*) Rr114 (*0.039*)	D7R13 (*0.001*)

The *p*-values associated with each of the two tests (Fdist2 [43] and DetSel [44], [45] analyses) for each of the four population pairs are reported for the two MHC-linked loci: Msat-Tnf and D20Img2. The supposedly neutral microsatellites for which a significant result was obtained in the analyses are also indicated, together with their *p*-values (in the case of Fdist2 analysis, all the presumed neutral loci revealing significant had F_ST_ higher than expected).

**Figure 2:**
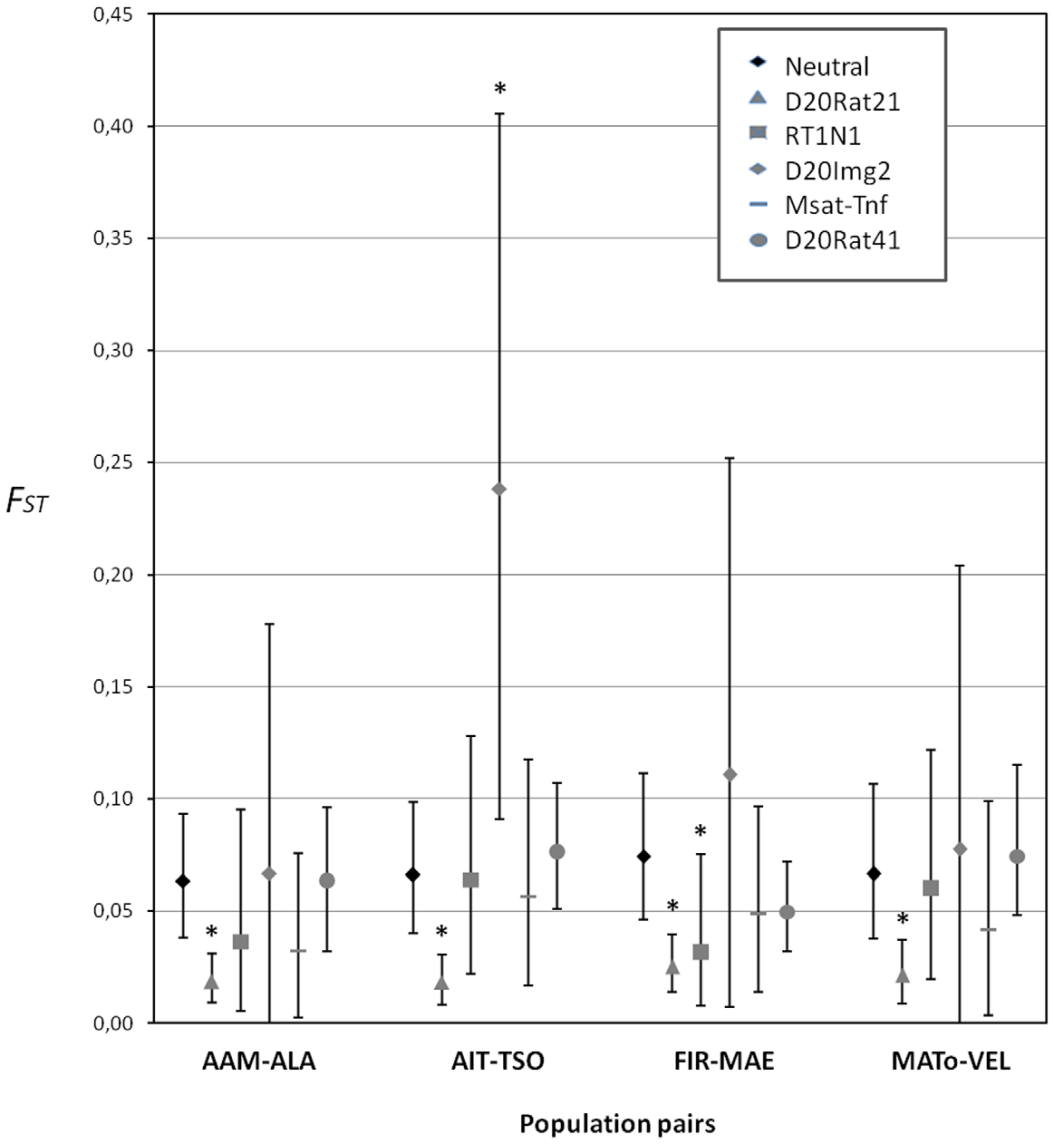
Comparison of pairwise population differentiation levels (*F_ST_*) between the five MHC-linked microsatellites and the neutral loci for the Madagascar's dataset. The points correspond to the mean *F_ST_* obtained over 1000 bootstrap and the error bars to the 95% confidence intervals (determined .from the 25^th^ and 975^th^ value in the ranked list of 1000 bootstrap *F_ST_* values). The neutral values are estimates from the global dataset of 13 presumed-neutral microsatellites. * indicate the MHC-linked markers significantly differing from neutral microsatellites for *F_ST_* estimates.

The pairwise *F_ST_* comparisons carried out between MHC-linked microsatellites (either duplicated or not) and neutral loci (global *F_ST_*) within each population pair of the Madagascar dataset are presented in Figure 2. Higher *F_ST_* was obtained for and the locus D20Img2 in the population pair AIT-TSO (*p*-value obtained through bootstrapping over individuals: *p* = 0.007), in accordance with the results obtained with the simulation-based programs (see above). The duplicated loci D20Rat21 had significantly lower *F_ST_* values than neutral markers in the four population pairs (*p* = 0.001 for AAM-ALA, *p* = 0 for AIT-TSO, *p* = 0.001 for FIR-MAE and *p* = 0.005 for MATo-VEL). Finally, the duplicated locus RT1N1 had low genetic differentiation estimate only in the population pair FIR-MAE (*p* = 0.033).

### Detecting signatures of selection for the Betafo dataset

For the Betafo dataset, the two non-duplicated MHC-linked microsatellites had overall *F_ST_* values within the envelope expected under neutrality, by Beaumont's method [43] (Fdist2 program, data not shown) and were therefore not identified as being subject to natural selection (*p* = 0.260 for Msat-Tnf and *p* = 0.338 for D20Img2). However, two presumed neutral microsatellite loci were found to be subject to purifying selection: Rr017 (*p* = 0.037) and Rr054 (*p* = 0.040). Significant isolation by distance was observed for neutral markers (*p* = 0.005). Partial Mantel tests with neutral microsatellite variation kept constant showed a positive effect of geographic distance on D20Rat41 (r^2^ = 12.9; *p* = 0.003), RT1N1 (r^2^ = 16.8; *p* = 0.001), D20Img2 (r^2^ = 10.4; *p* = 0.006) and Msat-Tnf (r^2^ = 11.4; *p* = 0.005) (Table 4). By contrast, the duplicated locus D20Rat21 presented no significant evidence of isolation by distance (Table 4) and its mean *F_ST_* value (0.014±0.008) indicated weaker genetic differentiation than for neutral loci (95% CI = (0.05; 0.07)).

**Table 4:** Partial Mantel tests carried out on the Betafo dataset for each of the five MHC-linked microsatellites.

Locus	Model	Partial Mantel	
		R^2^ (%)	P
**D20Rat21**	Global model	5.13	0.1906
	Geography	0	0.3978
	Neutral	2.43	0.155
	Geography*Neutral	0.93	
**RT1N1**	Global model	17.05	**0.0029**
	Geography	16.77	**0.001**
	Neutral	0	0.8457
	Geography*Neutral	0.25	
**D20Img2**	Global model	12.18	**0.0162**
	Geography	10.38	**0.0063**
	Neutral	3.78	0.1134
	Geography*Neutral	−1.99	
**Msat-Tnf**	Global model	14.14	**0.008**
	Geography	11.45	**0.0049**
	Neutral	0.91	0.4627
	Geography*Neutral	1.76	
**D20Rat41**	Global model	22.12	**0.0008**
	Geography	12.87	**0.0034**
	Neutral	5.15	0.0674
	Geography*Neutral	4.06	

## Discussion

In this study, we developed five MHC-linked microsatellites from the three classes of the MHC gene family in *R. rattus*. No linkage disequilibrium was found between the two non duplicated MHC-linked loci, Msat-Tnf and D20Img2 (this analysis was not possible for the duplicated loci), which are about 500 kb apart within the *R. norvegicus* genome. The MHC is characterized by islands of strong linkage disequilibrium, interspersed with recombination hotspots. Many factors, such as the selective and demographic history of the populations, may have a major effect on recombination patterns [46]. The absence of significant linkage disequilibrium between MHC loci was not surprising, as the islands of strong linkage disequilibrium have been estimated to cover about 50 kb in the human MHC class II region [47], and recombination hotspots appear to be largely conserved between mammalian species [48]. Our results show that different selective pressures affected the MHC-linked microsatellites studied, as frequently reported in previous studies, even for markers from the same MHC class (see for example [10], [11], [49], [50]).

As parasite-mediated selection is thought to be the driving force behind MHC evolution, pathogens (rather than other non pathogenic selective pressures) may account for the selection signatures of MHC-linked markers [6]. In Madagascar, plague is the only known highly virulent pathogen capable of differentiating between the *R. rattus* populations of the highlands and lowland areas [51]. We therefore have good reason to think that the selection signatures detected for the MHC-linked markers in this study will enable us to identify valuable candidate genes for plague resistance.

Firm evidence was obtained for natural selection acting on the D20Img2 locus, which presented levels of differentiation higher than expected under neutrality in some population pairs of the Madagascar dataset, whether in analyses based on two simulation-based programs (Fdist2 or DetSel) or using an empirical test. A few presumed neutral loci also displayed significant levels of differentiation (Table 3), but D20Img2 was more frequently detected as an outlier. This pattern may be interpreted as a signal of directional selection between the plague focus and the plague-free zone, as expected in conditions of plague-mediated selective pressure. We therefore expected D20Img2 to display a lower level of differentiation than neutral markers within the Betafo dataset, in which every population was assumed to be subject to plague selection. Instead, we showed an effect of geographic location on both loci, after controlling for demographic and stochastic factors. This geographic pattern within the plague focus may reflect spatial heterogeneity in plague selective pressures between *R. rattus* populations and the occurrence of spatially localized epizootics and epidemics. This would be consistent with the spatial clustering of human plague cases reported in the Betafo region, and the high variability of plague seroprevalence (between 0% and 44% [52]) in the rat populations studied. Spatial heterogeneity in plague selection pressure may also account for the detection of selection signatures in only some of the population pairs of the Madagascar dataset: those at sites within the plague focus at which human plague cases had been recorded in the last ten years (AIT in 2003 and AAM in 1998; database of the Malagasy Health Ministry). Alternatively, the genetic diversity of plague bacteria may imply geographic variation in the type of selective pressure. Indeed, a recent study was able to detect genetic diversity in plague bacterial strains isolated from patients in Madagascar [53]. Our four central highlands populations in the Madagascar dataset are located in regions where isolated strains were different; and even at the small spatial scale of the region investigated for the Betafo dataset, at least two plague genotypes were found. These different strains would however consist in different selective pressure only if they not only vary for neutral loci but also in their infection phenotype, but the latter was not investigated to date.

For the other four MHC-linked markers, the evidence for a selection signature were either weak or selection was less clearly related to plague. Indeed, an effect of geographic location, after controlling for demographic and stochastic factors, was also found for D20Rat41, Msat-Tnf and RT1N1. However, as no selection signature was obtained for these markers in the Madagascar dataset, the geographic pattern observed may not be specifically plague-mediated. Instead, it may be accounted for by the spatial variation of other selective pressures within the studied area of the plague focus, probably mediated by unidentified pathogens. The lack of a clear selective signal on these MHC markers does not however imply that there is no plague-mediated selection acting on the related MHC regions. Indeed, the main drawback of the MHC-linked microsatellite approach lies in the low reliability of negative results as an absence of selection signal may only be due to a low linkage disequilibrium between the gene under selection and the studied microsatellite. Finally, the low *F_ST_* estimates found in the duplicated locus D20Rat21 for both the Madagascar and Betafo datasets probably result from balancing selection [40]. The low genetic differentiation between plague focus and plague-free zone suggests similar selective pressures in these two regions. Consequently, balancing selection acting on D20Rat21 could most likely be exerted by undetermined pathogens homogeneously distributed in Madagascar.

The intensity of plague-mediated natural selection observed in this study did not depend on the distance between microsatellite markers and identified MHC candidate genes. The best *a priori* candidate among the five markers investigated was Msat-Tnf, because this marker was the closest to an identified gene (230 bp from *Tnf* in the *R. norvegicus* genome) and various studies have shown that the cytokine TNF-α plays an important role in host defense against plague (see introduction). However, this marker showed no clear evidence of plague-mediated selection. By contrast, the D20Img2 locus, for which stronger evidence of plague-mediated selection was obtained with both datasets, was located further from any known MHC gene in the *R. norvegicus* genome. The closest MHC gene to D20Img2 was Ddr1 (epithelial Discoidin domain receptor 1, 42 kb from D20Img2), which has no known function in defense against pathogens. The Ier3 (Immediate early response 3, located about 77 kb from D20Img2) gene, which encodes a protein involved in T-cell proliferation (according to the Ensembl database) and apoptosis [54], is the most probable best candidate for pathogen-mediated natural selection in the vicinity of D20Img2.

In terms of the selection mechanisms acting on MHC-linked loci, we found no evidence of heterozygote advantage, as non duplicated loci (D20Img2 and Msat-Tnf) were at Hardy-Weinberg equilibrium. As in other studies (reviewed in [6]), it remains possible that the selection of heterozygotes is not strong enough to be detected within a single generation [3]. Spatial variation in selective pressure was observed for all MHC-linked loci except D20Rat21, through geographic effects in the Betafo dataset and the high *F_ST_* for D20Img2 found within the population pairs in the Madagascar dataset. Plague-specific seroprevalence levels differed markedly within the plague focus region studied (see above), and a fortiori between the plague focus and the plague-free zone. Unfortunately, comparing allelic frequencies between seroprevalence categories within each population was not possible here because the sample size was too small given the number of alleles. However, this type of analysis would improve characterization of the spatial variability of plague-mediated selective pressure. Consequently, as in other studies reporting an effect of geographic location on the spatial genetic structure of MHC genes [11], [12], [55] or higher differentiation than for neutral loci [9], [11], our study highlights the need to characterize the variability of pathogen exposure more accurately, for evaluation of the contribution of pathogen-mediated fluctuating selection to these patterns.

## Materials and Methods

### Rat sampling

Field sampling was carried out systematically by joint teams comprising staff from the IRD (Institut de Recherche pour le Développement), from the IPM (Institut Pasteur de Madagascar) and from the Malagasy Ministry of Health (Communicable Disease Control Department). Each trapping campaign was validated by the national, regional and local health authorities. Rats were caught alive in wire-mesh and Sherman traps placed within houses and outdoors, according to a previously described protocol [56]. Outside houses, traps were set with the agreement of the village head, and always on the edge of cultivated fields, so as not to cause any damage in crops. Within houses, traps were set after the approval of the owner or tenant of the house. The only sampled rodents were introduced rodents (house mouse, black rat and Norway rat), which are classified as harmful and with no protected status in Madagascar. No permission was therefore required for their capture.

Animals were euthanized by cervical dislocation once trapped (as previously recommended [57]). Madagascar has no ethics committee that oversees animal experimentation, and the IRD has no ethics board to review animal experimentation protocols. However, the ANR-SEST (Agence Nationale pour la Recherche, Santé-Environnement et Santé-Travail) program on plague diffusion, which partially funded this project, has been approved by the managing director of the IRD. Additionally, regional approval was obtained from the regional head of veterinary service (Hérault, France), for the rodent sampling, euthanasia and tissue harvesting (approval no. B 34-169-1) carried out during this study. An autopsy was carried out and a piece of ear or tail was stored in 95% ethanol for genetic analyses.

The Madagascar dataset consisted of four pairs of populations, each consisting of one population from the plague focus and one from the plague-free zone, separated by a distance of 103 to 140 km (Figure 1, Table 1). These populations were sampled between 1996 and 2007, but each population pair was sampled in less time (maximum 4 years, Table 1) so that temporal variation is unlikely to drastically affect our results. The Betafo dataset consisted of 12 populations from seven villages sampled between 2006 and 2007 within an area of 150 km^2^ close to the town of Betafo, within the plague focus. In this area, the most recent human plague cases were reported in 2004. At least 14 rats were trapped at each study site (see Table 1 for sample sizes).

### MHC-linked microsatellite loci

The MHC-linked microsatellites used in this study on *R. rattus* were inferred from *R. norvegicus* genome. Indeed, the two species only diverged 2.9My ago [58], and the gene syntheny was shown to be extremely high between both species [59]. Consequently, it appears safe to assume that locations of the studied microsatellites are the same in *R. rattus* as in *R. norvegicus*. We obtained specific primers for five MHC-linked microsatellites (Table 2). Previously published primers, designed for use in *R. norvegicus*, were used for the D20Img2 locus [60]. We used the *R. norvegicus* genome sequence to design primers for the D20Rat21 locus. Finally, the primers used for the three other loci (D20Rat41, RT1.N1 and Msat-Tnf) were designed into two steps: amplification of the *R. rattus* sequences with *R. norvegicus* primers, followed by sequencing and the design of primers more specific to *R. rattus*, based on the sequences obtained. The distance between each MHC-linked microsatellite and the closest known MHC gene was between 300 and 42,000 bp in *R. norvegicus* (Table 2).

### Molecular biology methods

Genomic DNA was extracted from rat tissues with the DNeasy® Tissue Kit (Qiagen), according to the manufacturer's instructions. Two final elutions, each in 50 µl of AE buffer were carried out.

We genotyped 13 presumed neutral microsatellites as previously described [56], [61]. All these loci are polymorphic (5–20 alleles per population) dinucleotide microsatellites.

The MHC-linked microsatellites were amplified in two multiplex PCRs: one for Msat-Tnf and RT1N1 (PCR1) and the other for D20Rat41, D20Rat21 and D20Img2 (PCR2). Both PCRs were performed in a final volume of 10 µl, containing 0.2 µl of each primer, 5 µl of multiplex premix (Qiagen) and DNA (2 µl for PCR1, and 1 µl for PCR2). The two PCRs were performed according to the same program: initial denaturation (15 min at 95°C), followed by 35 cycles of denaturation (30 s at 94°C), hybridization (90 s at 60°C) and elongation (60 s at 72°C), and a final extension (30 min at 72°C). All PCR products for MHC-linked microsatellites were pooled together in a single run, and were detected with an automated ABI Prism 3130XL sequencer (Applied Biosystems). Electrophoretic gels were read with GeneMapper 3.7 (Applied Biosystems). Stuttering was relatively low in duplicated loci, and profiles were mostly clear. Each individual with an ambiguous profile was however re-amplified once by simple PCR (to avoid primer competition), and considered as a null genotype when ambiguity remains (less than 4% of the genotyped individuals).

### Data analysis

For non-duplicated MHC-linked and presumed neutral loci, we checked for Hardy-Weinberg equilibrium and genotypic linkage disequilibrum between pairs of loci with Genepop v. 4 [62]. In both cases, we corrected for multiple testing by the false discovery rate (FDR) approach, with Q-value software (available from http://genomics.princeton.edu/storeylab/qvalue/); the tuning parameter λ was fixed at 0 [63]. As some loci exhibited heterozygote deficiencies, we used Micro-checker 2.2.3 [64] to evaluate whether it could be explained by the occurrence of null alleles. We then used the software Freena (INRA Montpellier website, available www.montpellier.inra.fr/URLB, accessed 2012 February 7) [42] to verify that mean null allele frequencies were low at these loci, as high null alleles frequencies can impact genetic differentiation estimations [42].

Under the hypothesis of overdominance (or heterozygote advantage, see introduction), an excess of heterozygotes would be expected at MHC loci. We thus tested for Hardy-Weinberg disequilibrium (H1: heterozygote excess) within each studied population for the two non duplicated MHC-linked loci using Genepop program. The FDR approach was also used to correct for multiple testing.

For the “Madagascar” dataset, we expected the level of genetic differentiation to be higher for MHC-linked microsatellites than for neutral loci within population pairs. We evaluated the deviation from neutrality for all non-duplicated MHC-linked and presumed neutral microsatellites, using two approaches for the detection of selection, based on the *F_ST_* values for each of the four population pairs. We first used the method of Beaumont & Nichols [43], as implemented in Fdist2 (available at http://www.rubic.rdg.ac.uk/~mab/software.html). This approach uses an island model and simulates the distribution of *F_ST_* conditioned on heterozygosity, under the null hypothesis of drift and migration only. We carried out 50,000 simulations of two demes, assuming a stepwise mutation model. We then used the Vitalis et al. [44] method, implemented in DetSel software [45]. This method performs coalescent simulations under a divergence model in which an ancestral population splits into two isolated populations. The simulations were performed with a stepwise mutation model and a mean mutation rate μ = 0.0005. We used several different values for ancestral population size (*N*_e_ = 1,000, 10,000, 100,000), and the time since divergence was set at 1,200 generations (because *R. rattus* probably colonized the central highlands about 700 years ago [65]). For each pairwise analysis, we performed a total of 1,000,000 simulations, with each set of nuisance parameter values representing a third of the total simulations. We applied a minimum allele frequency of 0.01 to the observed and simulated data.

We then used a method similar to the one described in Neff & Fraser [66] to empirically compare the pairwise *F_ST_* value for each MHC-linked (duplicated and non-duplicated) locus with the global *F*_ST_ for the neutral loci within the four population pairs. For each of the five MHC-linked markers, as well as for the global neutral dataset (13 loci), 1000 datasets were obtained by bootstrapping over individuals. For all the datasets, pairwise *F*_ST_ were computed within each population pair from allelic frequencies (see also [12], [55]) using the package Arlecore from Arlequin v 3.5 software [67]. Note that allelic frequencies cannot be directly estimated for the duplicated microsatellites (data available only consist in allele presence/absence, genotypes may be unknown). Allelic frequencies were thus inferred from the number of individuals carrying a given allele divided by the total number of alleles observed in a population (see also [12], [55]). The proportion of bootstrap replicates in which the *F*_ST_ of MHC-linked markers were higher or lower than neutral *F*_ST_ was used as *p*-value for the null hypothesis that one of MHC-linked markers had higher or lower *F*_ST_ than the neutral markers (see also [66]).

For the “Betafo” dataset, we expected the level of genetic differentiation to be lower for MHC-linked microsatellites than for presumed neutral loci, provided that the selection pressure exerted by plague was uniform over the whole area [40]. We tested this hypothesis for non duplicated microsatellites, with Fdist2 (with the same parameters as for the analysis of the Madagascar dataset, see above). We did not use DetSel on the “Betafo” dataset: it does not conform to the model of a single ancestral population diverging into two isolated populations with no migration between them, as it corresponds to a small scale metapopulation sampling design.

A significant isolation by distance (IBD) pattern was found for presumed neutral microsatellites within the “Betafo” dataset (as already reported [52]). For each MHC-linked locus, we performed partial Mantel tests [68], which evaluated the correlative link between the pairwise *F_ST_* at the MHC locus and geographic distance while keeping constant differentiation at neutral microsatellites (excluding those identified by FDist2 as corresponding to selection signature in the “Betafo” dataset). Under the hypothesis of strong selection pressures acting on MHC loci, we would expect a lower isolation by distance pattern than under neutrality for these loci. Pairwise *F_ST_* values were estimated for each locus with Genepop v. 4 for non duplicated loci, or from allelic frequencies for duplicated loci. Partial Mantel tests were performed with Fstat v. 2.9.3.2 [69], with 20,000 permutations.

## Supporting Information

Figure S1 **Selection signatures detected with Beaumont & Nichols's approach (Fdist2 program** [**43**] **) applied to the Madagascar dataset and the 15 loci: Msat-Tnf, D20Img2 and the 13 apparently neutral microsatellites.** The analysis was performed for the four population pairs, each consisting of one population from the plague focus and one population form the plague-free zone. Genetic differentiation between the two populations (*F_ST_*) is plotted against heterozygosity. Median and 95% confidence interval are indicated with solid lines, whereas the 99% confidence interval is indicated with a dotted line.
(PDF)

Figure S2 **Results of the test of natural selection for Msat-Tnf, carried out with the method of Vitalis et al.** (DetSel program [44], [45]) for the four population pairs studied for the Madagascar dataset. Genetic differentiation for the first population (*F_1_*) is plotted against genetic differentiation for the second population (*F_2_*).
(PDF)

Figure S3 **Results of the test of natural selection for D20Img2, carried out with the method of Vitalis et al.** (DetSel program [44], [45]) for the four population pairs studied for the Madagascar dataset. Genetic differentiation for the first population (*F_1_*) is plotted against genetic differentiation for the second population (*F_2_*).
(PDF)

Table S1 **Null allele frequencies estimates obtained using FreeNA software** [**42**] **for each of the 15 non duplicated microsatellite loci in the 20 populations.**
(PDF)
