# Supplementary figures and images for: Contrasted Patterns of Selection on MHC-Linked Microsatellites in Natural Populations of the Malagasy Plague Reservoir
Source: PLoS One. 2012 Mar 5;7(3):e32814. doi: 10.1371/journal.pone.0032814 (PMC3293896; doi:10.1371/journal.pone.0032814)

AAM - ALA

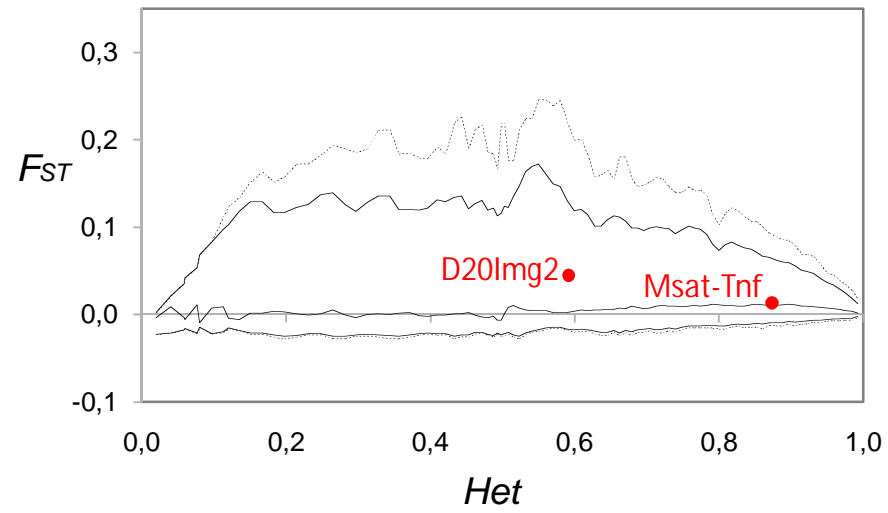

AIT - TSO

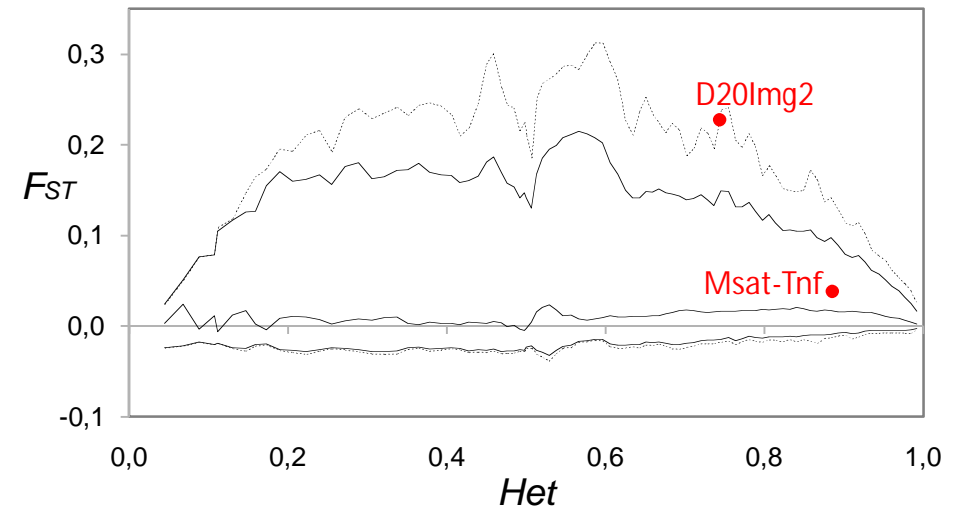

FIR - MAE

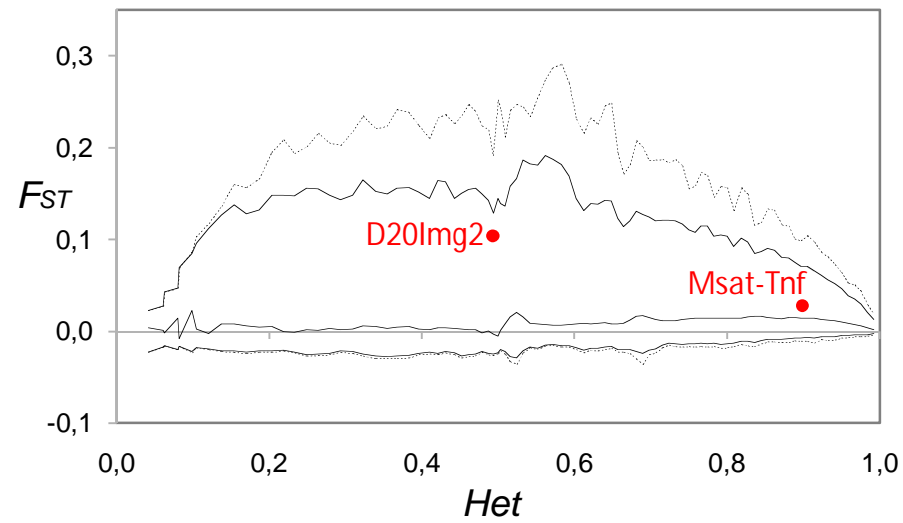

MATo - VEL

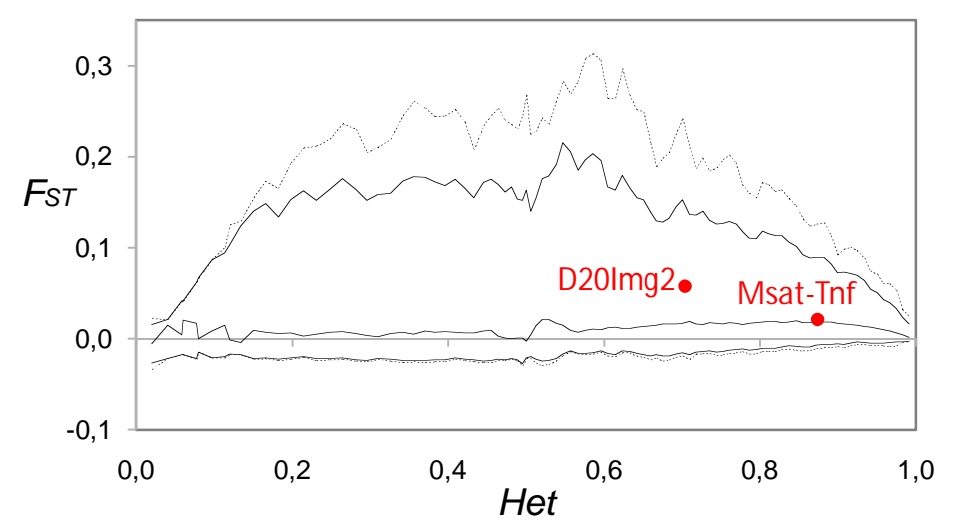

Supplement: Figure S1 — Selection signatures detected with Beaumont & Nichols's approach (Fdist2 program [43] ) applied to the Madagascar dataset and the 15 loci: Msat-Tnf, D20Img2 and the 13 apparently neutral microsatellites. The analysis was performed for the four population pairs, each consisting of one population from the plague focus and one population form the plague-free zone. Genetic differentiation between the two populations (FST) is plotted against heterozygosity. Median and 95% confidence interval are indicated with solid lines, whereas the 99% confidence interval is indicated with a dotted line. (PDF) [file pone.0032814.s001.pdf]

**TNF / AAM-ALA**

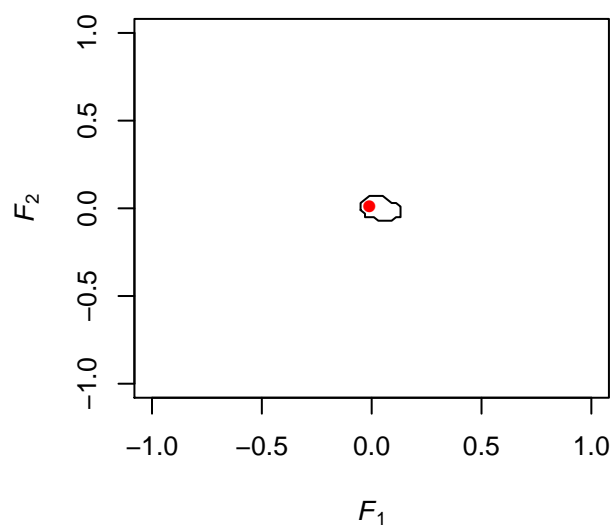

**TNF / AIT-TSO**

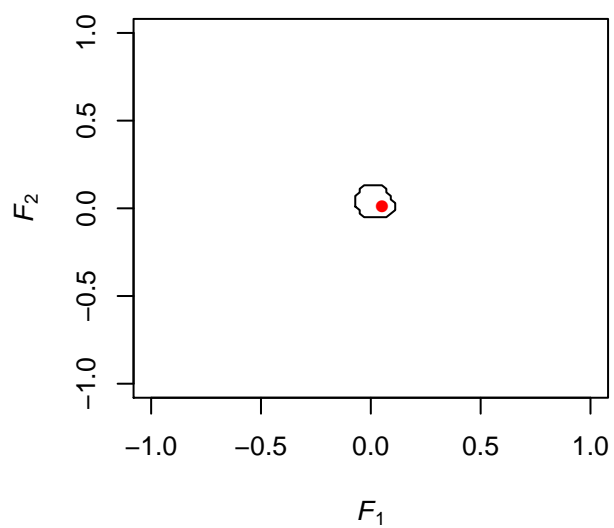

**TNF / FIR-MAE**

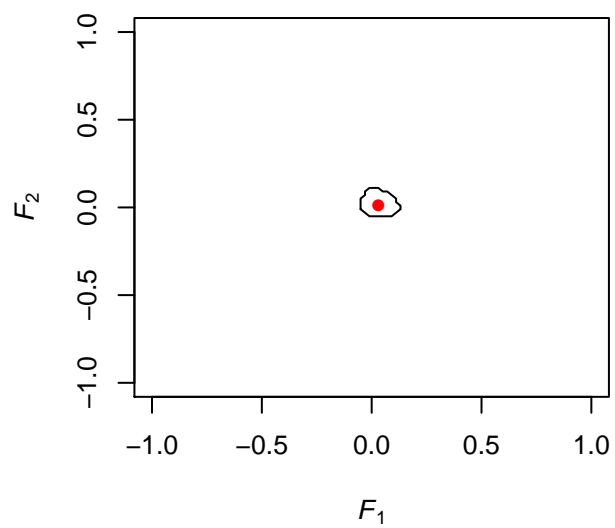

**TNF / MAtO-VEL**

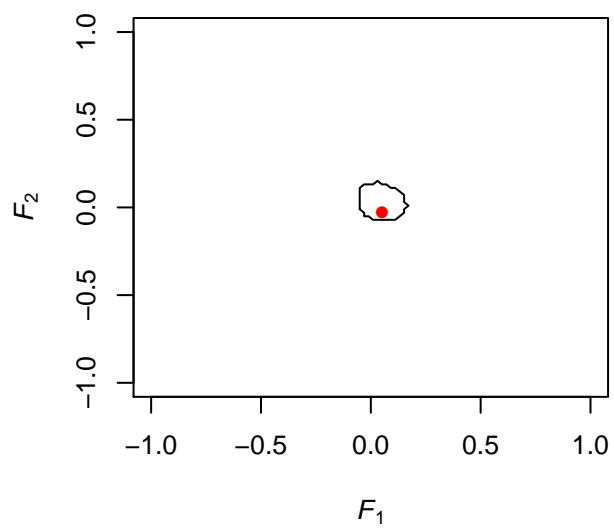

Supplement: Figure S2 — Results of the test of natural selection for Msat-Tnf, carried out with the method of Vitalis et al. (DetSel program [44], [45]) for the four population pairs studied for the Madagascar dataset. Genetic differentiation for the first population (F1) is plotted against genetic differentiation for the second population (F2). (PDF) [file pone.0032814.s002.pdf]

**D20lmg2 / AAM-ALA**

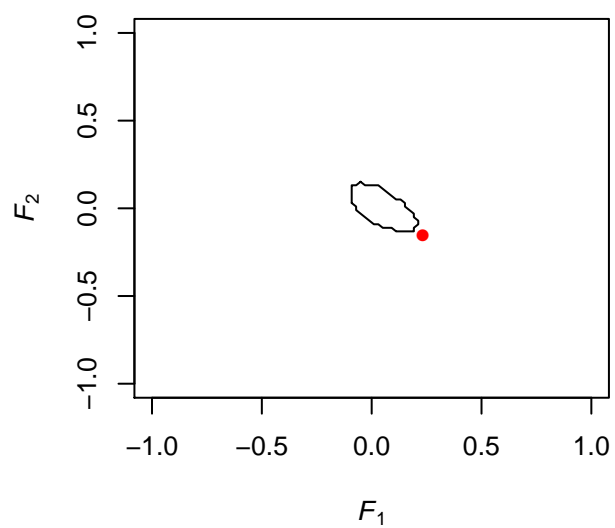

**D20lmg2 / AIT-TSO**

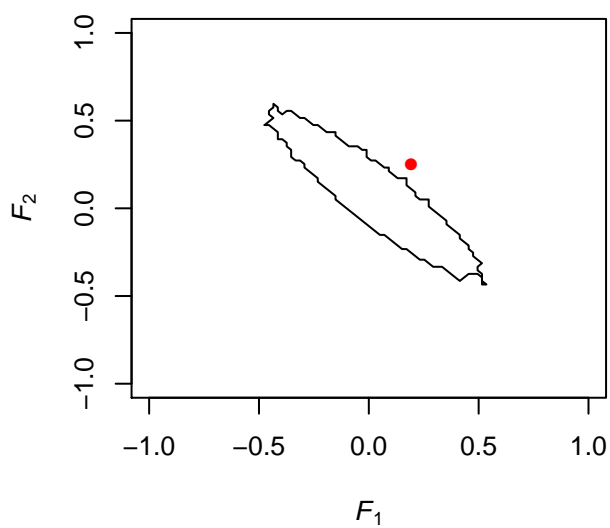

**D20lmg2 / FIR-MAE**

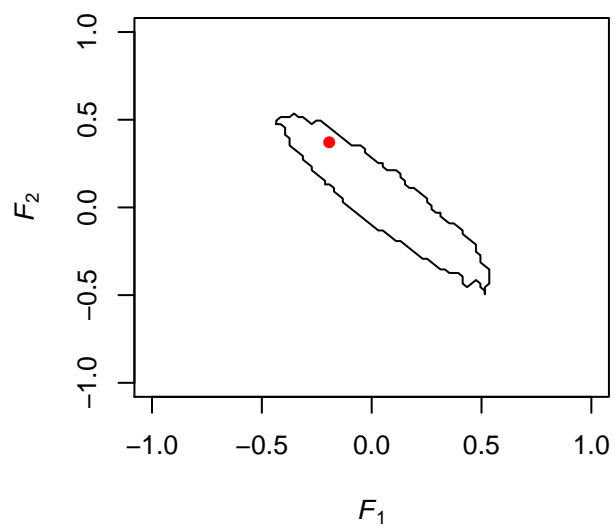

**D20lmg2 / MAto-VEL**

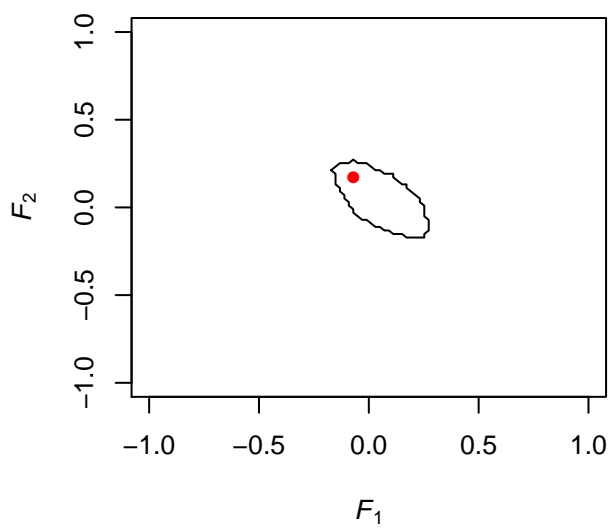

Supplement: Figure S3 — Results of the test of natural selection for D20Img2, carried out with the method of Vitalis et al. (DetSel program [44], [45]) for the four population pairs studied for the Madagascar dataset. Genetic differentiation for the first population (F1) is plotted against genetic differentiation for the second population (F2). (PDF) [file pone.0032814.s003.pdf]
