# Supplementary material for: Contrasted Patterns of Selection on MHC-Linked Microsatellites in Natural Populations of the Malagasy Plague Reservoir
Source: PLoS One. 2012 Mar 5;7(3):e32814. doi: 10.1371/journal.pone.0032814 (PMC3293896; doi:10.1371/journal.pone.0032814)
Supplement: Table S1 — Null allele frequencies estimates obtained using FreeNA software [42] for each of the 15 non duplicated microsatellite loci in the 20 populations. (PDF) [file pone.0032814.s004.pdf]

Population		Presumed neutral microsatellites													MHC-linked loci	
		Rr014	Rr017	Rr022	Rr054	Rr067	Rr068	Rr093	Rr107	Rr114	D10R20	D11R56	D7R13	D11M5	D20Img2	TNF
Madagascar dataset	AAM	0,013	0,066	0,000	0,026	0,148	0,000	0,016	0,000	0,000	0,026	0,000	0,043	0,000	0,177	0,058
	ALA	0,000	0,000	0,000	0,000	0,000	0,000	0,044	0,000	0,036	0,099	0,027	0,000	0,006	0,025	0,000
	AIT	0,000	0,000	0,020	0,000	0,020	0,000	0,089	0,000	0,102	0,041	0,000	0,000	0,073	0,000	0,000
	TSO	0,000	0,101	0,000	0,000	0,143	0,000	0,000	0,019	0,000	0,106	0,205	0,121	0,121	0,000	0,000
	FIR	0,000	0,084	0,000	0,000	0,124	0,000	0,000	0,036	0,003	0,000	0,000	0,000	0,000	0,000	0,071
	MAE	0,015	0,000	0,000	0,000	0,000	0,000	0,000	0,000	0,000	0,068	0,000	0,010	0,000	0,000	0,013
	MAto	0,000	0,000	0,036	0,007	0,093	0,000	0,000	0,000	0,000	0,000	0,000	0,021	0,000	0,079	0,023
	VEL	0,000	0,000	0,041	0,000	0,104	0,000	0,000	0,000	0,000	0,000	0,145	0,000	0,000	0,000	0,000
Betafo dataset	ApgE	0,000	0,099	0,000	0,000	0,000	0,033	0,000	0,000	0,000	0,032	0,059	0,044	0,109	0,000	0,000
	ApgM	0,044	0,000	0,057	0,000	0,001	0,079	0,000	0,011	0,105	0,148	0,000	0,000	0,114	0,087	0,065
	AlnE	0,000	0,000	0,000	0,000	0,046	0,000	0,000	0,000	0,067	0,000	0,045	0,000	0,000	0,072	0,000
	AlnM	0,000	0,000	0,000	0,000	0,072	0,000	0,000	0,051	0,000	0,095	0,035	0,000	0,017	0,084	0,000
	AtnE	0,046	0,000	0,047	0,008	0,000	0,000	0,000	0,014	0,000	0,247	0,000	0,000	0,024	0,000	0,026
	AhyM	0,053	0,038	0,038	0,134	0,151	0,000	0,191	0,000	0,007	0,000	0,098	0,000	0,010	0,015	0,041
	VniE	0,000	0,000	0,000	0,000	0,000	0,000	0,000	0,000	0,000	0,000	0,202	0,000	0,009	0,000	0,000
	VniM	0,013	0,009	0,079	0,000	0,094	0,046	0,076	0,068	0,092	0,000	0,000	0,000	0,000	0,000	0,000
	MkfE	0,016	0,125	0,000	0,000	0,000	0,000	0,009	0,000	0,112	0,000	0,000	0,133	0,012	0,044	0,000
	MkfM	0,029	0,046	0,002	0,000	0,000	0,194	0,000	0,045	0,000	0,000	0,067	0,000	0,080	0,036	0,031
	AklE	0,000	0,015	0,000	0,000	0,000	0,024	0,000	0,000	0,094	0,000	0,000	0,000	0,000	0,127	0,000
	AklM	0,000	0,063	0,000	0,000	0,000	0,000	0,059	0,000	0,169	0,000	0,000	0,000	0,000	0,035	0,185
Mean over 20 populations		0,011 ± 0,018	0,032 ± 0,042	0,016 ± 0,024	0,009 ± 0,03	0,050 ± 0,059	0,019 ± 0,046	0,024 ± 0,048	0,012 ± 0,021	0,039 ± 0,054	0,043 ± 0,066	0,044 ± 0,067	0,019 ± 0,039	0,029 ± 0,043	0,039 ± 0,05	0,026 ± 0,044
